# Spinal Region Prevalence of Angle Trunk Rotation in Adolescent Male Soccer Players: A Cross-Sectional Study

**DOI:** 10.3390/jfmk10020134

**Published:** 2025-04-16

**Authors:** Eleni Theodorou, Eleanna Chalari, Marios Hadjicharalambous

**Affiliations:** 1 Human Performance Laboratory, Department of Life Sciences, School of Life and Health Sciences, University of Nicosia, P.O. Box 24005, 1700 Nicosia, Cyprus; elenitheodorou@hotmail.com; 2 Department of Sport and Physical Education, Faculty of Health Sciences, Aegean College, 105 64 Athens, Greece; e.chalari@aegeancollege.gr

**Keywords:** spinal asymmetries, youth soccer players, region prevalence

## Abstract

**Background:** Spinal asymmetries and postural deviations are common concerns in young athletes, particularly those engaged in sports requiring repetitive and asymmetrical movements. Soccer, as a dynamic sport, involves high levels of trunk rotation, rapid directional changes, and frequent loading asymmetry, which may predispose players to spinal deviations. This study aimed to investigate the regional prevalence of primary and secondary angle trunk rotation (ATR) in adolescent male soccer players across different age groups. **Methods:** A total of 502 male participants (291 soccer players and 211 nonathletes) aged 11 to 14 years were included in the study. Participants underwent scoliosis screening using a scoliometer following Adam’s forward bending test. ATR measurements were recorded at the thoracic and lumbar spinal regions, with primary ATR (ATR-A) and secondary ATR (ATR-B) assessed separately. A chi-square test (χ^2^) evaluated the association between training level and the regional prevalence of ATR across different age groups. **Results:** No significant differences were observed in the regional prevalence of ATR-A and ATR-B in the 11-year-old group. However, by age 12, significant differences emerged in ATR-A prevalence (χ^2^(3) = 16.469, *p* = 0.001), while ATR-B remained nonsignificant (χ^2^(2) = 4.040, *p* = 0.133). In the 13- and 14-year-old groups, significant associations were found for both ATR-A (χ^2^(3) = 57.219, *p* < 0.001; χ^2^(4) = 34.157, *p* < 0.001) and ATR-B (χ^2^(3) = 31.481, *p* < 0.001; χ^2^(2) = 17.805, *p* < 0.001), with moderate to strong effect sizes. **Conclusions:** While no significant differences were observed in younger players, 13- and 14-year-old soccer players exhibited a significantly higher prevalence of ATR than nonathletes. The findings suggest that soccer players exhibited a higher prevalence of spinal asymmetries, particularly in older age groups, with lumbar ATR being more pronounced. The study highlights an increasing trend of spinal asymmetries with training level among young soccer players, likely due to the cumulative effects of asymmetric movement patterns. These findings emphasize the need for early detection and corrective interventions to mitigate potential long-term musculoskeletal imbalances in adolescent soccer players.

## 1. Introduction

The impact of sports on spinal health has been a subject of increasing interest in sports medicine and orthopedics [1,2,3]. Sports such as swimming and running tend to have a lower risk of postural disturbances in the cervical, thoracic, and lumbar regions of the spine [4]; snowboarding and horseback riding present a high incidence of traumatic lumbar spine injuries [5]; and soccer [4,6,7,8], basketball, and volleyball [9] may have a detrimental impact on body posture, potentially increasing postural deviations over time and having long-lasting effects on players’ spinal health [10]. In soccer, for example, repetitive activities such as heading the ball have been linked to degenerative changes in the cervical spine, while lumbar strain is common due to the extensive sprinting and sudden directional changes involved in the game [11]. Additionally, professional soccer players have been found to develop asymptomatic degenerative changes in their lumbar spine particularly, which may lead to chronic issues later in their careers [12].

Participation in sports plays a crucial role in the physical development of young athletes [7,13]. However, the high-intensity demands of the daily training and early specialization of athletic training may particularly influence musculoskeletal adaptations, leading to potentially negative effects on spinal health [1,14] causing spinal injuries and low back pain (LBP) [15,16]. The lifetime prevalence, for example, of LBP in adolescents ranges between 18% and 51% [17,18]. Among young athletes, soccer players in particular are subjected to high levels of repetitive strain and asymmetrical movements, which may produce spinal asymmetries such as scoliosis, kyphosis, and lordosis [7]. Recent evidence, for example, suggests that spinal asymmetries may impact neuromuscular performance, flexibility, and injury susceptibility in young soccer players, and asymmetries in spinal posture were found to reduce lower-body explosive power and flexibility, particularly in athletes with kyphotic or scoliotic postures [1].

Moreover, LBP during adolescence is predictive of chronic pain and musculoskeletal dysfunction in adulthood, emphasizing the long-term implications of early spinal stress [19,20]. Youth athletes often experience a higher incidence of lumbar spine injuries, including spondylolysis, disc degeneration, and stress fractures, which may remain undetected because of underreporting or lack of early screening [21,22]. Given that adolescence is a critical period for skeletal development, the cumulative impact of training loads and sport-specific asymmetries may impose substantial strain on the spine leading potentially to altered postural alignment and functional limitations [23]. Consequently, understanding the epidemiology of spinal conditions in this population is essential for informing targeted preventive strategies and guiding evidence-based training protocols in youth sports [2].

Furthermore, variations in spinal asymmetries and related musculoskeletal adaptations differ across sports due to the unique biomechanical demands imposed on athletes. Soccer players, for example, demonstrate higher incidences of interlimb strength [24] and muscular asymmetries, which have both been associated with increased injury risk. Lower limbs muscle asymmetries [25] have also been widely recognized as key factors in producing postural deviations and increasing injury risk in soccer players. Recent studies have adopted objective methods such as tensiomyography (TMG) to assess interlimb asymmetries and neuromuscular imbalances. For instance, Buoite Stella et al. (2022) identified significant lower-limb muscle asymmetries in male soccer players using TMG, showing a link between these asymmetries and performance in countermovement jumps, highlighting their potential impact on neuromuscular performance and injury prevention strategies [25]. Similarly, Espada et al. (2023) evaluated both unilateral and bilateral strength asymmetries in high-level male soccer players, emphasizing that pronounced asymmetries may persist even at professional level necessitating targeted intervention [26].

In children and youth soccer players, a potential reason for the development of spinal asymmetries and LBP might be the disturbance of ATR-A and ATR-B, due perhaps to early sports-specialization exposure [7]. In a previous study from our laboratory, the association of the dominant hand (DH) and leg (DL) with the side of the ATR-A in boys and girls during their growth was examined [6]. It was found that girls had higher measurements for the ATR than boys, but cross-laterality, in primary and secondary ATR, was found only in boys, of whom the majority specialized in soccer [6]. In a subsequent study, we examined the association between DL and the contralateral side of functional scoliosis, as well as whether any of the postural asymmetries’ evaluated variables may be a reliable predictor of functional scoliosis development in young male soccer players. We observed that leg dominance in youth soccer players may be a factor causing truncal rotation on the contralateral side, which may progressively lead to functional scoliosis [7]. Given these findings, it is essential to elaborately examine the prevalence of spinal asymmetries in youth soccer players, where asymmetric loading and frequent directional changes may predispose players to spinal imbalances. The aim, therefore, of the present study was to examine the association between training level and the regional prevalence of ATR in soccer players vs. nonathletes in four age subgroups (11-, 12-, 13-, and 14-year-olds). It was hypothesized that ATR, in the lumbar region particularly, would be more prevalent among soccer players than the nonathlete control groups across all age categories.

## 2. Materials and Methods

### 2.1. Participants

A total of 502 male children participated in this study, including 291 soccer players (mean age: 13 ± 2 years; average height: 158 ± 17.5 cm; average weight: 50.6 ± 12 kg) and 211 nonathletic participants (control group) with a comparable mean age of 13 ± 2 years, height of 158.3 ± 11 cm, and weight of 50.5 ± 21 kg. All participants volunteered for the study. Both soccer players and control participants were further divided into four age-specific subgroups: 11, 12, 13, and 14 years. Prior to the scoliosis screening, the experimental procedures were explained, and written consent was obtained from the participants’ parents. None of the participants reported any illness or musculoskeletal injury at the time of the study. Ethical approval was granted by the National Bioethics Committee (EEBK/EP/23/8/2021.01.169), and the research adhered to the ethical principles outlined in the Declaration of Helsinki by the World Medical Association.

### 2.2. Inclusion and Exclusion Criteria

Only male schoolchildren were eligible for participation in the study. The control group consisted exclusively of nonathletic participants. The experimental group included boys from soccer academies who actively engaged in organized soccer training from the age of seven and below, and soccer players were required to have a minimum of at least three years of continuous training experience. Both groups, soccer players and nonathletes, were recruited from three different big cities of the country. Participants in the soccer group who reported any illness or musculoskeletal injury at the time of the study were excluded. Additionally, although written informed consent was obtained from all parents or legal guardians, any child who expressed unwillingness to participate was respectfully excluded, in full adherence to ethical research principles.

### 2.3. Experimental Design

The height and weight of all participants were measured. Scoliosis screening was conducted at the training facilities for soccer players and at the school for nonathletes. Coaches oversaw the process for athletes, while a teacher was present for nonathletes. Parents were also allowed to be present during the measurements.

### 2.4. Scoliosis Screening

A qualified kinesiologist measured truncal rotation using a scoliometer (Mizuho Osi^®^, Mizuho OSI Inc., Tokyo, Japan) [27,28]. The assessment began with the participant performing Adam’s forward bending test, initially bending forward with extended arms touching the knees for thoracic and thoracolumbar measurements while keeping the feet together. Afterward, the participant bent forward with extended arms pointing downward for lumbar measurements, maintaining feet together [27,28]. During the test, when the researcher observed any spinal asymmetry, they placed the scoliometer at the identified level and region to obtain measurements [27,28]. The recorded data included scoliometer readings, the convexity side, and the spinal region of truncal rotation. If asymmetry was present at two different regions, both measurements were recorded, with the higher value classified as the ATR-A and the lower as the ATR-B. Participants were categorized into four subgroups based on their chronological age: 11-, 12-, 13-, and 14-year-olds.

### 2.5. Statistical Analysis

According to the normality test (Kolmogorov–Smirnov), the variables violated normality assumptions. Descriptive results were reported as the median and interquartile range (IQR). A chi-square test (χ^2^) of independence was conducted to determine whether the physical activity level was associated with the region of primary and secondary ATR across both groups. To assess the strength of these associations, Cramér’s V effect size (ES) was calculated, with values interpreted based on Cohen’s criteria: small (0.1), medium (0.3), and large (0.5) ES. Statistical significance was set at *p* < 0.05 [29]. All statistical analyses were conducted using the SPSS software (version 30 for Windows; IBM SPSS Inc., Chicago, IL, USA).

## 3. Results

### 3.1. Intraclass Correlation Coefficient

The results for the intratester reliability of the inclinometer and scoliometer demonstrated excellent reproducibility (Table 1).

### 3.2. Descriptive Characteristics of the Participants

Table 2 displays the physical characteristics of the groups across different age categories. The ATR-A and ATR-B values represent mean ± standard deviation for descriptive purposes only.

### 3.3. Chi-Square (χ^2^)

**Figure 1:**
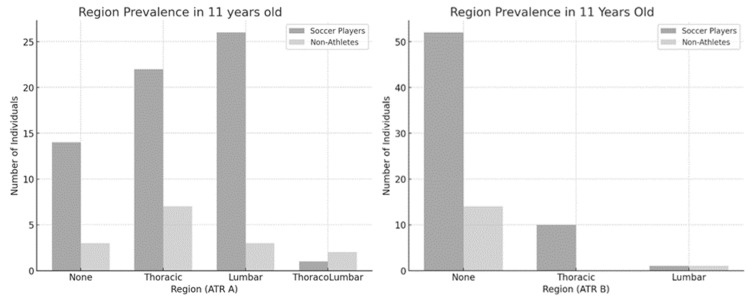
Regional prevalence between soccer players and nonathletes (11 years old). ATR-A (**left**); ATR-B (**right**).

In the 11-year-old group (Figure 1), no significant association was observed between physical activity level and the regional prevalence of ATR-A [χ^2^(3) = 6.297, *p* = 0.098, ES = 0.284] and ATR-B [χ^2^(2) = 3.767, *p* = 0.152, ES = 0.220]. Although there was no statistical significance in the comparison between 11-year-old soccer players and nonathletes in primary ATR, the results revealed a clear trend. Soccer players consistently outnumbered nonathletes in all regions. The thoracic and lumbar regions had the highest representation, where soccer players significantly exceeded nonathletes. Therefore, a trend of a spinal asymmetry appeared to initiate at the age of eleven.

**Figure 2:**
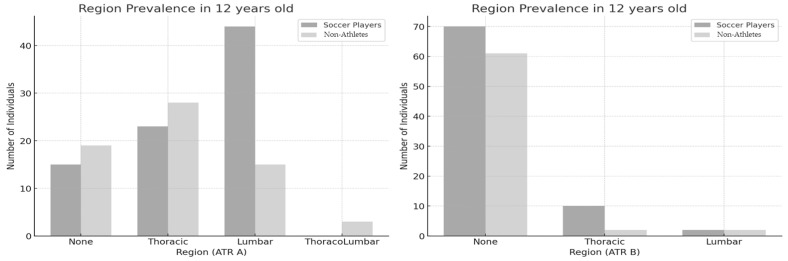
Regional prevalence between soccer players and nonathletes (12 years old). ATR-A (**left**); ATR-B (**right**).

In the 12-year-old group (Figure 2), a significant association was found between physical activity level and the regional prevalence of ATR-A, χ^2^(3) = 16.469, *p* = 0.001, ES = 0.335. However, in the same group, no significant association was observed between physical activity level and the regional prevalence of ATR-B, χ^2^(2) = 4.040, *p* = 0.133, ES = 0.166. In respect to primary ATR, the thoracic region had the highest number nonathletes. However, the lumbar region had the highest number of soccer players, significantly exceeding nonathletes, indicating a strong preference for physical activity among those categorized in the lumbar region.

**Figure 3:**
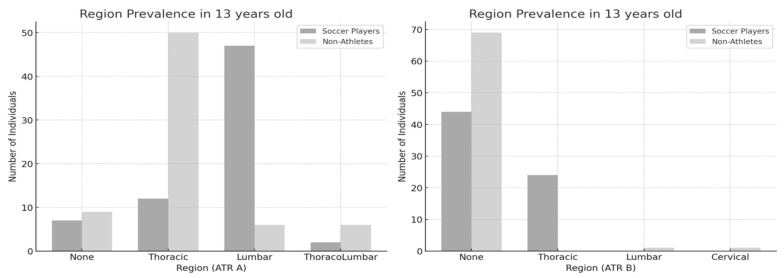
Region prevalence between soccer players and nonathletes (13 years old). ATR-A (**left**); ATR-B (**right**).

**Figure 4:**
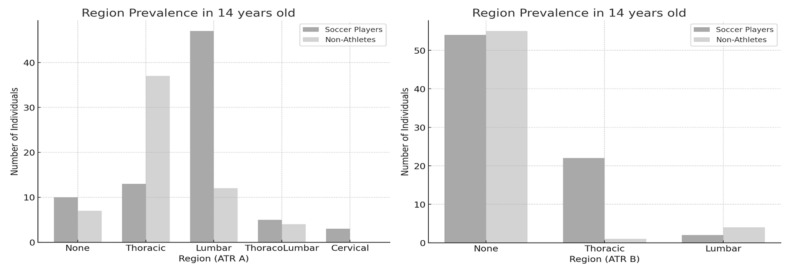
Region prevalence between soccer players and nonathletes (14 years old). ATR-A (**left**); ATR-B (**right**).

In the 13-year-old group (Figure 3), a significant association was found between physical activity level and the regional prevalence of ATR-A [χ^2^(3) = 57.219, *p* < 0.001, ES = 0.642] and ATR-B [χ^2^(3) = 31.481, *p* < 0.001, ES = 0.476]. In the 14-year-old group (Figure 4), a significant association was found between physical activity level and the regional prevalence of ATR-A [χ^2^(4) = 34.157, *p* < 0.001, ES = 0.498] and ATR-B [χ^2^(2) = 17.805, *p* < 0.001, ES = 0.359]. The trend in primary ATR observed in the 12-year-old group became increasingly evident in the older age groups. Among nonathletes, the thoracic region was the most commonly affected, whereas among soccer players, the lumbar region was predominantly involved. Additionally, a thoracic asymmetry appeared to develop as a secondary ATR in soccer players.

## 4. Discussion

The present study aimed to investigate the association between training level and the regional prevalence of ATR-A and ATR-B among adolescent male soccer players across four different age groups (11-, 12-, 13-, and 14-year-olds). The findings revealed significant variations in ATR region prevalence with increasing age, indicating a potential negative association between prolonged engagement in soccer and spinal asymmetries. It is important to emphasize that our statistical approach focused on categorical differences in the distribution of asymmetry regions—not on comparing raw ATR angle values between groups. The significant associations identified through chi-square tests highlighted a shift in asymmetry localization that corresponded with training level rather than a difference in absolute degrees of trunk rotation. These results highlight the importance of monitoring spinal health in youth athletes and implementing targeted interventions to address potential postural deviations in an attempt to secure spinal health and avoid future chronic LBP.

It is noted that LBP represents a significant global health burden and is increasingly prevalent among adolescents, with estimates suggesting that up to 30% of teenagers experience recurrent episodes by mid-adolescence [30]. This early onset of postural and musculoskeletal disorders has been linked to factors such as physical inactivity, prolonged sedentary behavior, and, conversely, repetitive strain from sport-specific movements. In youth athletes, particularly those involved in sports characterized by asymmetric and high-load actions such as soccer, the risk of developing postural abnormalities and chronic LBP is amplified. The growing prevalence of lumbar spine issues in the adolescent population, which was examined in the current study, not only affects current physical performance but predisposes individuals to chronic LBP in adulthood, with associated socioeconomic costs and reduced quality of life [31].

Consequently, numerous studies have investigated the impact of sports, particularly soccer, on postural alignment and body symmetry in young athletes. Postural deviations, including ATR, serve as key indicators of spinal asymmetry and scoliosis, which may develop or progress with age, especially in youth athletes participating in unilateral or asymmetric sports. The findings of this study indicate that while no significant differences in ATR regional prevalence were detected in younger participants (ages 11 and 12), statistically significant differences emerged in older age groups (ages 13 and 14). As they matured, soccer players exhibited a higher occurrence of ATR in both measured regions (ATR-A and ATR-B) than nonathletes, a trend consistent with prior research [8,32,33].

Soccer is characterized by unilateral movement patterns that impose asymmetric loads on the body, particularly affecting the lower limbs and trunk [34]. This imbalance likely contributes to the elevated ATR values observed in soccer players [35]. Kalata et al. (2020) reported that asymmetrical sports, such as soccer, are associated with greater bilateral strength asymmetry, potentially leading to postural deviations over time [36]. In a previous study, it was found that 13- and 14-year-old soccer players exhibited a higher prevalence of primary ATR-A in specific spinal regions, likely reflecting the cumulative effects of these unilateral stresses. In contrast, younger players (ages 11 and 12) did not show significant differences, suggesting that their bodies still possess a strong capacity to adapt to asymmetric loads [8]. However, as children grow, these asymmetries become more pronounced and increasingly difficult to correct without targeting interventions [2].

Lourenço et al. [33] reported notable postural deviations in older soccer players, particularly in shoulder and pelvic alignment, while younger players exhibited no significant irregularities. Their study emphasized that the impact of asymmetric loading patterns intensifies with age [33]. These changes accelerate during early adolescence, potentially worsening pre-existing asymmetries [37]. The rapid increases in height and shifts in body composition during growth spurts heighten the risk of postural deviations, particularly in sports that involve fast, asymmetric movements, such as soccer [38]. The growing differences in ATR region prevalence among 13- and 14-year-old soccer players observed in this study may be attributed to the physical changes that accompany puberty and growth spurts, which are characteristic of this age group [38]. The significantly higher ATR prevalence in older soccer players suggests that as they grow taller, the asymmetries resulting from repetitive soccer movements become more pronounced. This aligns with previous research indicating that as soccer players mature, they are more prone to developing postural asymmetries, particularly scoliotic and kyphotic postures, which can lead to decreased flexibility and neuromuscular performance [1,8].

The results of this study align with existing literature [1,6,39], indicating that young soccer players face a heightened risk of developing spinal asymmetries due to the repetitive and asymmetric movements inherent in the sport. The lack of statistically significant differences in ATR regional prevalence among 11-year-old soccer players and nonathletes suggests that early exposure to soccer training does not have an immediate impact on spinal asymmetry. However, the increased prevalence of thoracic and lumbar ATR in soccer players compared with nonathletes suggests the emergence of postural imbalances over time. These findings are consistent with those of Theodorou et al. (2022), who reported that spinal asymmetries in young soccer players negatively affected neuromuscular performance, reducing flexibility and strength [1]. In a subsequent study, we found that leg dominance may be a factor causing truncal rotation on the contralateral side, which may progressively lead to functional scoliosis in youth soccer players [7]. Unfortunately, in the current study, we did not examine the effect of the DL specifically on muscular size and symmetry, muscular symmetrical strength, balance, flexibility, or injury rate. Apart, therefore, from the hypothesis that leg dominance may induce postural asymmetries as a result of early specialization, it is important to point out that functional and structural spine asymmetries attributed to limb dominance may also negatively influence muscle strength, power, and flexibility. Previous studies, for example, assumed the existence of between-limb asymmetry secondary to the repetitive unilateral nature of kicking the ball [40], and interlimb asymmetries in strength, power, balance, flexibility, and electromyographic muscle activity have been considered as possible factors associated with both performance and injury risk in sports [41]. Consequently, future research should perhaps focus on simultaneously examining the effect of postural asymmetries and DL on muscular size and symmetry, muscular symmetrical strength, balance, flexibility, and injury rate.

By age 12, a significant association between physical activity and ATR region prevalence emerged, suggesting that the cumulative effects of soccer training begin to manifest as spinal asymmetries. The predominance of lumbar ATR in soccer players highlights the lower back as a particularly vulnerable region of the spine. These results are supported by Júnior et al. (2020), who reported a higher prevalence of lumbar spine lesions in young soccer players than in nonathletes [42]. The asymmetrical demands placed on the body during soccer training likely contribute to this regional disparity.

The significant differences in ATR prevalence among 13- and 14-year-old soccer players reinforce the hypothesis that prolonged exposure to asymmetric movements in soccer contributes to postural deviations. The findings indicate that thoracic ATR is more prevalent in nonathletes, whereas lumbar ATR is more pronounced in soccer players. This suggests that soccer training predominantly influences lumbar rotation, likely via the frequent kicking and pivoting movements required in the sport. Theodorou et al. (2024) found similar results, demonstrating that leg dominance plays a crucial role in truncal rotation asymmetries [7]. The observed secondary ATR trends, particularly the increased prevalence of thoracic rotation in soccer players, support the notion that compensatory mechanisms develop as players adapt to sport-specific demands. This aligns with research by Sannicandro et al., which showed that soccer training, particularly small-sided games, significantly increased lower limb asymmetry, likely leading to trunk compensations [43]. Furthermore, it was suggested that targeted corrective exercise interventions can mitigate these asymmetries, emphasizing the potential benefits of early detection and intervention [2].

The current results suggest that long-term participation in soccer training contributes to spinal asymmetries, particularly in the lumbar region. Given that such asymmetries may increase the risk of musculoskeletal injuries and impair athletic performance, integrating preventive strategies into training programs is crucial. As previously demonstrated, strength and asymmetry performance are significantly influenced by competitive level [44] and age [7,8], indicating that higher-level players may be more susceptible to postural deviations [44]. Overall, this study reinforces the growing body of evidence suggesting that soccer training influences spinal asymmetry development, particularly in the lumbar region. Coaches and medical professionals should consider integrating targeted interventions to address these asymmetries early, reducing their potential long-term impact on players’ musculoskeletal health and performance.

## 5. Conclusions

The findings of this study suggest that adolescent soccer players, of the ages of 13 and 14 years particularly, exhibited a higher regional prevalence of spinal asymmetries (ATR-A and ATR-B) than nonathletes of the same age. These differences were most notable in the lumbar region, indicating a sport-specific pattern of asymmetrical spinal loading potentially attributable to the demands of daily soccer practice. While no significant differences were observed in younger age groups (11 and 12 years), the emergence of asymmetry in older athletes supports the hypothesis that cumulative exposure to asymmetric movement patterns contributes to postural adaptations over time. These findings underscore the importance of early postural screening and suggest that targeted intervention strategies may be warranted to mitigate the potential long-term impact of sport-specific asymmetries in youth soccer players. Future research should explore: (a) the longitudinal trends in spinal asymmetries, evaluating the effectiveness of corrective training programs in minimizing postural imbalances in adolescent soccer players; (b) the simultaneous evaluation of the effect of postural asymmetries and DL on muscular size and symmetry, muscular symmetrical strength, balance, flexibility, and injury rate; and (c) biomechanical analysis of movement patterns for providing deeper insights into the mechanisms driving asymmetry development in young athletes.

## Figures and Tables

**Table 1:** Intraclass correlation coefficients.

	Intraclass Correlation Coefficient (ICC)	95% Confidence Interval (CI)	*p*-Value
Scoliometer	0.988	0.981–0.993	0.001 *
Inclinometer T1–T2	0.987	0.979–0.993	0.001 *
Inclinometer T12–L1	0.995	0.991–0.997	0.001 *
Inclinometer S2–S3	0.995	0.991–0.997	0.001 *

Spinous process of: T1 = first thoracic vertebra, T2 = second thoracic vertebra, T12 = twelfth thoracic vertebra, L1 = first lumbar vertebra, S2 = second sacral vertebra, S3 = third sacral vertebra. * = statistically significant difference.

**Table 2:** Soccer players and nonathletes characteristics per age category.

Groups	Subgroups	Height (cm)	Weight (kg)	ATR-A (°)	ATR-B (°)
**Soccer Players (n = 291)**	11 yrs (n = 63)	147 ± 6	42 ± 8	2 ± 3	0 ± 0
	12 yrs (n = 82)	153 ± 11	47 ± 10	2 ± 1	0 ± 0
	13 yrs (n = 68)	163 ± 16	54 ± 12	3 ± 2	0 ± 3
	14 yrs (n = 78)	168 ± 12	59 ± 11	3 ± 2	0 ± 2
**Nonathletes** **(n = 211)**	11 yrs (n = 15)	146 ± 7	41 ± 11	2 ± 2	0 ± 0
	12 yrs (n = 65)	152 ± 8	45 ± 5	2 ± 3	0 ± 0
	13 yrs (n = 71)	157 ± 10	54 ± 9	2 ± 2	0 ± 0
	14 yrs (n = 60)	167 ± 7	59 ± 15	3 ± 2	0 ± 0

## Data Availability

The data presented in the current study are available from the corresponding author upon request.

## Abbreviations

The following abbreviations are used in this manuscript:

IQR	Interquartile range
ES	Effect size
ATR	Angle trunk rotation
ATR-A	Primary angle trunk rotation
ATR-B	Secondary angle trunk rotation
